# The application of wide-field laser ophthalmoscopy in fundus examination before myopic refractive surgery

**DOI:** 10.1186/s12886-017-0647-4

**Published:** 2017-12-15

**Authors:** Lin Liu, Fang Wang, Ding Xu, Chunlei Xie, Jun Zou

**Affiliations:** Department of Ophthalmology, Shanghai Tenth People’s Hospital, Tong Ji University, Shanghai, 200072 China

**Keywords:** Wide-field laser ophthalmoscope, Refractive surgery, Optomap panoramic 200Tx

## Abstract

**Background:**

To evaluate wide-field laser ophthalmoscopy (Optomap 200Tx) for screening retinal lesions before myopic refractive surgery.

**Methods:**

Seventy-eight eyes of 78 consecutive refractive surgery candidates were included in this study. All subjects underwent Optomap 200Tx, mydriatic slit-lamp lens examination and the Goldmann three-mirror contact lens examination, which was considered as the reference method for determining retinal lesions.

**Results:**

Forty of 78 eyes had retinal lesions (51.28%) and three eyes had retinal breaks (3.85%), which were diagnosed by the Goldmann three-mirror contact lens examination. Compared to the Goldmann three-mirror contact lens examination, the detection rate with the Optomap 200Tx was 91.73%% for retinal lesions, while the detection rate of mydriatic slit-lamp lens exams was 81.20%. There were no statistically significant differences among the three methods used for the diagnoses of myopic conus, tessellation and retinal breaks(all *p* > 0.05). For peripheral retinal lesions, the detection rate of the Optomap 200Tx examinations were similar to the Goldmann three-mirror contact lens exams (all *p* > 0.05), but were higher than the results of slit-lamp lens examinations (all *p* < 0.05). Regarding the vitreoretinal adhesions, the Goldmann three-mirror contact lens examinations had higher detection rates than did the Optomap 200Tx examinations (*p* = 0.031).

**Conclusions:**

The Optomap 200Tx examinations is a convenient and feasible method to determine fundus pathological changes in myopic patients, especially for patients who can not endure pupil dilation. In order to avoid misdiagnosis of peripheral retinal lesions, Goldmann three-mirror contact lens examination is needed.

## Background

Myopia is the most common type of refractive error. The prevalence of myopia has continued to increase, especially in Asian countries [1, 2]. Laser refractive surgery has been performed for decades. There have been tremendous advancements in terms of technique and technology, making laser refractive surgery increasingly precise and highly predictable, and laser in situ keratomileusis (LASIK) has become increasingly popular for the treatment of myopia over the past decades [3, 4].

With increased axial elongation, myopia may lead to peripheral retinal lesions, such as retinal breaks, chorioretinal atrophy, Fuch’s spot, lacquer cracks, pigmentary degeneration, lattice degeneration, posterior staphyloma, and white without pressure symptoms [5]. The association of myopic LASIK with rhegmatogenous retinal detachment (RRD) remains controversial and the frequency of RRD after LASIK was reported to be 0.06% to 0.25% [6–8]. The lattice degeneration, a risk factor for retinal breaks, may not progress into RRD by prophylactic laser photocoagulation before the refractive surgery. Post-LASIK RRD usually occurs in eyes with complex retinal breaks [9, 10]. Therefore, before the refractive surgery, a thorough retinal examination is very important.

The purpose of this study was to compare the diagnostic properties of the non-mydriatic Optomap ultra-wide-field scanning laser ophthalmoscope (SLO) with the mydriatic slit-lamp lens [90 diopters (D)] and the Goldmann three-mirror contact lens examination for myopic screening.

## Methods

### Subjects

Seventy-eight eyes of 78 consecutive refractive surgery candidates were recruited for the study. The median age was 26 years (range, 21–39 years). The average spherical equivalent (SE) was −5.50 D (SD, 3.00 D with a range of −1.0 D to −10.75 D), and the axial length was 24.47–28.49 mm (average, 25.68 ± 2.10 mm). None had a history of ocular surgery, ocular trauma, or systemic or ocular health abnormality. The study was approved by the Ethics Committee of the Shanghai Tenth People’s Hospital, and written informed consent was obtained by all the patients in this study.

Each subject underwent a complete ophthalmology examination, which included measurements of visual acuity, refraction, and intraocular pressure (IOP) by a noncontact tonometer. Axial length measurements were obtained in each eye with the IOL Master (Carl Zeiss Meditec, Inc., Dublin, CA, USA) and by stereoscopic fundus examination. The stereoscopic fundus examination was performed by three retinal specialists using ultra-wide-field scanning laser ophthalmoscopy (Optomap 200Tx™, Optos®, Marlborough, MA, USA), mydriatic slit-lamp biomicroscopy (90 D lens), and a mydriatic Goldmann three-mirror contact lens. The three retinal specialists were blinded to the clinical data.

### Optomap imaging

The Optomap Panoramic 200Tx device is a SLO with two scanning laser wavelengths of green (532 nm) and red (633 nm). Optomap imaging was performed without pupil dilation. Several images were taken and the best image of each eye was saved for grading. It took 0.25 s to obtain one image by SLO. The total scanning time was 3–5 min, including patient positioning. All Optomap imaging was performed by the same clinician, the four directions (Temporal, Nasal, Superior and Inferior) guiding and normotopia for each eye have been photographed and recorded.

### Statistical analysis

Statistical analyses were performed with commercially available software (SPSS, version 15.0; SPSS Inc., Chicago, IL, USA). The results of the three types of fundus examinations were compared by the paired chi-square test. A *p* value < 0.05 was considered statistically significant.

## Results

### Goldmann three-mirror contact lens examination

Forty of 78 eyes had retinal lesions (51.28%), which were diagnosed by the Goldmann three-mirror contact lens examination. Twenty eyes (25.64%) were detected with myopic conus in 78 eyes, twenty-four eyes (30.77%) had varying degrees of tessellation, thirty-four eyes (43.59%) had white without pressure, eighteen eyes (23.08%) had lattice degeneration, twenty-seven eyes (34.62%) had cream sample and pigment degeneration, three eyes (3.85%) had retinal breaks, and seven eyes (8.97%) had vitreoretinal adhesions.

### Optomap 200TX imaging

In 78 eyes of 78 myopia patients, Twenty eyes (25.64%) were detected with myopic conus, twenty-four eyes (30.77%) had varying degrees of tessellation, thirty-two eyes (41.03%) had white without pressure, sixteen eyes (20.51%) showed lattice degeneration, twenty-five eyes (32.05%) exhibited cream sample and pigment degeneration, three eyes (3.85%) had retinal breaks, and two eyes (2.56%) had vitreoretinal adhesions.

### Mydriatic slit-lamp lens examination (90 D)

Twenty eyes (25.64%) were detected with myopic conusin in 78 eyes, twenty-four eyes (30.77%) had varying degrees of tessellation, twenty-eight eyes (35.90%) showed white without pressure, ten eyes (12.82%) had lattice degeneration, Twenty eyes (25.64%) had cream sample and pigment degeneration, two eyes (2.56%) had retinal breaks, and four eyes (5.13%) had vitreoretinal adhesions.

### Comparisons of the three methods

Compared to the Goldmann three-mirror contact lens examination, the detection rate of the Optomap 200Tx for retinal lesions was 91.73% while the detection rate of the mydriatic slit-lamp lens exam was 81.20%. There were no statistically significant differences among the three methods for the diagnosis of myopic conus, tessellation retinal break. For peripheral retinal lesions, the results of the Optomap 200Tx examinations were similar with the Goldmann three-mirror contact lens examinations(all *p* > 0.05), but was better than the results of the slit-lamp lens examinations (all *p* < 0.05). Regarding vitreoretinal adhesion detection, the Goldmann three-mirror contact lens examination had a better performance than the Optomap 200Tx (*p* = 0.031). (Table 1).

**Table 1:** Comparisons of the three methods for detecting fundus lesions (paired chi-square test)

	Goldman three-mirror contact lens	Optomap 200TX	mydriatic slit-lamp lens (90 D)	P1	P2	P3
myopia conus	20	20	20	1.000	1.000	1.000
leopard fundus	24	24	24	1.000	1.000	1.000
white without pressure	34	32	28	0.727	0.040	0.031
lattice degeneration	18	16	10	0.754	0.008	0.031
cream sample and pigment degeneration	27	25	20	0.687	0.000	0.000
retinal break	3	3	2	1.000	1.000	1.000
vitreoretinal adhesions	7	2	4	0.031	0.375	0.375

P1, Goldman three-mirror contact lens and Optomap 200TX; P2, Goldman three-mirror contact lens and mydriatic slit-lamp lens; P3, Optomap 200TX and mydriatic slit-lamp lens (90 D); *P* < 0.05 was statistically significant. D, diopters

## Discussion

The role of LASIK as a potential additive risk factor for RRD in myopic eyes has been an issue of debate. The frequency of RRD after LASIK is reported to be 0.06% to 0.25% [6–8]. Arevalo et al. reported that the development of peripheral retinal tears or macular injury during LASIK was due to vitreous traction and deformity induced by anterior-posterior compression and expansion [11–14]. Therefore, examination of the vitreous and retinal before LASIK is necessary and should be a routine procedure.

The traditional retinal examination such as the Goldmann three-mirror contact lens examination always requires pupil dilation and corneal contacting, which could be uncomfortable and have a higher risk of infection. Recently, the ultra-wide-field scanning ophthalmoscope Optomap 200Tx has become commercially available. This device uses an ellipsoid mirror and can acquire a wide-field image of the fundus, with the Optomap 200Tx imaging system. It is now possible to scan 200 degree of the retina in a single photograph without pupil dilation and corneal contacting. This can provide a much larger image of the peripheral retina than the traditional Optomap 200 [15]. With the SLO, sharp images with high contrast are obtained and can be saved permanently. The Optomap 200Tx is widely accepted and be valuable for the evaluation of several retinal pathologies, including diabetic retinopathy, retinitis pigmentosa, uveitis, age-related macular degeneration and retinal detachment [16–22].

**Fig. 1:**
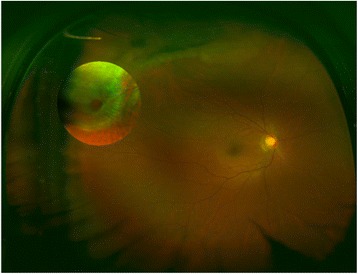
Peripheral retinal break with shallow detachment in myopia

Diagnostic imaging probably has become a significant supplement of traditional slit-lamp examination. Mackenzie et al. used the Optomap Panoramic 200 wide-field confocal scanning laser imaging system for detecting peripheral retinal lesions and found that the Optomap showed high specificity and moderate sensitivity for lesions posterior to the equator [21]. In current study, forty eyes (51.28%) of 78 eyes with retinal lesions and three eyes with retinal breaks (3.85%) were diagnosed by the Goldmann three-mirror contact lens examination.With the informed consent of patients, three patients who had retinal breaks underwent prophylactic laser photocoagulation to seal the breaks, even though they were asymptomatic. One of the three patients, who was peripheral retinal break with shallow detachment (Fig. 1), was performed advanced corneal surface ablation and the others were performed LASIK at least 2 weeks post-photocoagulation. The retinal detachment has not been occurred since the follow-up in this study. Compared with the Goldmann three-mirror contact lens examination, the detection rate of the Optomap 200Tx for retinal lesions was 91.73%, while the detection rate of the mydriatic slit-lamp lens examination was 81.20%.

In this study, higher detection rates were shown in the vitreoretinal adhesions in the Goldmann three-mirror contact lens examination compared with the Optomap 200Tx examination. Possible explanations are as the followings: Firstly, the stereoscopic sensing of the Optomap 200Tx for screening of retinal diseases was not as good as that of the Goldmann three-mirror contact lens examination. Moreover, the Optomap 200Tx provided a less real color image using two monochromatic red and green SLO scans that could be viewed separately or superimposed, resulting in a semi realistic bicolor Optomap fundus image.

In practical terms, it is more important to analyze the inconsistent results between the Goldmann three-mirror contact lens examination and the Optomap 200Tx. We found that the detection numbers of the peripheral retinal lesions using the Optomap 200Tx, such as the cream sample and pigment degeneration, seemed to be less than with the Goldmann three-mirror contact lens examination, although no statistical significance was reached. We suggest that this was mainly due to the examination being restricted by the eyelids and eyelashes, particularly for patients with hollow eyeballs and small palpebral fissures. These may lead to a smaller angle of view in the vertical direction. Therefore, for patients with hollow eyeballs and small palpebral fissures, the Goldmann three-mirror contact lens examination may be have some advantages.

## Conclusions

In conclusion, the Optomap 200Tx examinations is a convenient and feasible method for fundus pathological changes detection in myopic patients, especially for patients who can not endure pupil dilation.

## Abbreviations

AMD: Age-related macular degeneration
IOP: Intraocular pressure
LASIK: Laser in situ keratomileusis
RRD: Rhegmatogenous retinal detachment
SLO: Scanning laser ophthalmoscope
